# Factors associated with local failure after stereotactic radiation to the surgical bed of patients with a single breast cancer metastasis

**DOI:** 10.1007/s00701-025-06520-9

**Published:** 2025-04-22

**Authors:** Ory Haisraely, Marcia L. Jaffe, Yaacov R Lawrence, Zvi Symon, Anton Whol, Thaila Kaisman-Elbaz, Zvi R Cohen, Alicia Taliansky, Orit Kaidar-Person

**Affiliations:** 1 Radiation oncology department, Sheba Medical Center, Ramat Gan, Israel; 2 School of Medicine, Faculty of Medical and Health Science, Tel -Aviv University, Tel Aviv, Israel; 3 Neuro-Surgical Department, Sheba Medical Center, Ramat Gan, Israel; 4 Neuro-Oncology Unit, Sheba Medical Center, Ramat Gan, Israel; 5 GROW-School for Oncology and Developmental Biology, Maastricht University, Maastricht, The Netherlands

**Keywords:** Brain metastases, Breast cancer, Postoperative radiotherapy, Radiosurgery, Craniotomy

## Abstract

**Introduction:**

Breast cancer brain metastases (BCBM) are increasingly common due to improved systemic therapies prolonging survival. This study evaluates local control and factors influencing outcomes in patients with resected BCBM treated with postoperative stereotactic radiotherapy (SRT).

**Methods:**

A retrospective review included single resected BCBM treated with postoperative SRT from 2010 to 2022. The median follow-up was 28 months (range, 14–43). Variables analyzed included tumor size, biology, surgical corridor inclusion, radiation dose, and timing of SRT. Multivariable analysis was conducted using Cox regression.

**Results:**

62 patients were analyzed in multivariable analysis, HER2-positive status was associated with improved local control (HR: 0.76, 95% CI: 0.36–0.88, p = 0.032), as was a higher biologically effective dose (BED > 40 Gy, HR: 0.65, 95% CI: 0.45–0.89, p = 0.028). In contrast, tumor size > 5 cm (HR: 2.1, 95% CI: 1.7–4.6, p = 0.021) and delayed initiation of SRT beyond 28 days post-surgery (HR: 2.7, 95% CI: 1.9–4.7, p = 0.015) were associated with worse outcomes. Age, cystic metastases, inclusion of surgical corridor, and tumor location were not significantly related to local control. Radiation necrosis occurred in 13% of patients, predominantly asymptomatic.

**Conclusion:**

Postoperative SRT provides effective local control in resected BCBM. In multivariable analysis, HER2 positivity, higher BED, and timely SRT significantly influenced outcomes, while larger tumor size and delayed treatment were negative prognostic factors. Future research should optimize dosimetric strategies and integrate systemic therapy to improve local and intracranial control.

## Introduction

Metastatic breast cancer (mBC) is the second most common cancer associated with brain metastases [24]. As cancer research breakthroughs considerably improved survival of patients with breast cancer in the last decades, the incidence of brain metastasis is increasing accordingly [9, 40]. It has been suggested that breast cancer brain metastases (BCBM) occur more frequently among younger women, those with larger tumors or higher nuclear grade, in certain subtypes such as estrogen-receptor (ER)-negative and HER2 overexpressing tumors, and those with nodal metastases and lung metastasis [32]. The incidence of BCBM is increasing and is changing across breast cancer subtypes, and it is mainly attributed to success in controlling systemic disease and prolonged survival [22, 26]. An analysis done by Sperduto et al.,[35] showed that local therapy for BCBM has changed over 3 time periods between 1985 to 2017, mostly with lower use of whole brain radiation (WBRT) and increased use of stereotactic radiation, while craniotomy stayed stable within a range of 16–20% of the BCBM patients [35]. Surgery alone as a local therapy for brain metastases is associated with a very high local failure rate, reaching up to 70% within one year.[23, 28–30] One major trial and several retrospective studies showed that the addition of radiation after craniotomy improve local control compared to craniotomy alone, with better local control at the craniotomy site for WBRT compared to stereotactic radiation (SRT) to the surgical cavity, without improvement in overall survival [7, 20, 23, 28–30].

However, current guidelines from both the Society of Neuro-Oncology and the international SRT recommend radiation to the surgical cavity [33, 38]. Mainly due to fear of the toxicity from WBRT and lack of OS advantage with the addition of WBRT [5, 6, 20].

Sperduto et al.,[35], in their publication showed that prognosis of BCBM is highly dependent on factors such as tumor histology, molecular subtypes and number of brain metastasis, and the presence of systemic dissemination. In addition, local control (craniotomy site) after SRT was shown to be dependent on dose/fractionation and volume of the surgical cavity, site of resection, and timing of radiation in relation to craniotomy [1, 34]. However, none of the large, randomized trials of brain metastasis analyzed local control only in the BCBM population [20, 23, 28–30]. Grubb et al., [11] reported that the breast cancer molecular subtype might be a predictor for local control of BCBM treated with SRT. Suggesting a dose control relationship per BCBM subtype.

In this study we aim to evaluate the unique radio-sensitivity breast cancer brain metastasis according to dosimetry, tumor biology and clinical variable by assessing these factors influence on local control and distant intracranial failure.

## Methods

Institutional review board approval for a retrospective review was obtained. Inclusion criteria were all patients treated between 2012–2022 with SRT to the surgical cavity after craniotomy for a single brain metastases of breast cancer origin based upon pathology report of the brain metastasis. We excluded patients who had previously received radiation to the brain such as WBRT or preoperative SRT to brain metastasis or brain magnetic resonance imaging (MRI) and follow up after SRT. All patients in the analysis had undergone tumor board discussion with neurosurgeon, pathology, radiation oncology and radiology for the decision of post op radiotherapy. The dose regimen was determined by the primary radiation oncologist.

Background demographics, tumor related factors, pathologic and radiographic data, prior oncologic therapy, and detailed radiation data were extracted from the electronic medical record and from institutional radiotherapy databases. Disease-Specific (DS) Graded Prognostic Assessment (GPA) was determined based on medical records [35]. Systemic breast cancer status was defined using the European Society for Radiotherapy and Oncology (ESTRO) and European Organization for Research and Treatment of Cancer (EORTC) classification [11].

Primary breast cancer subtype was recorded according to tumor receptors, since subtype definition and receptor positivity (mainly Estrogen receptor, ER) changed according to guidelines, ER positive was regarded as positive immunohistochemistry staining (IHC) and systemic therapy included at any time point endocrine therapy. For the purpose of the analysis, we regarded ER positive, PR positive, HER2 negative as luminal -A like; ER positive, PR negative, HER2 negative – as luminal B like; HER 2 was considered positive if it had IHC + 3 staining or positive on ​Fluorescence In Situ Hybridization (FISH) and the systemic therapy included anti-HER2 at any time point.

The use of systemic therapy was also recorded. Systemic therapy initiated within three months of postoperative radiation was considered adjuvant.

Local failure was defined as tumor growth in the surgical cavity inside the radiation planning target volume using radiographic definition [23] and after tumor board discussion. Distant intracranial failure was defined as a new brain metastasis outside the surgical cavity. Time to failure was calculated from the completion of SRT. Toxicity was evaluated and graded by CTCAE v5.0 criteria.

### Statistical analysis

Descriptive analyses were performed using mean and SD for parametric variables and median with range for non-parametric variables. X^2^ test was used for categorical variables. Total dose (biological effective dose, BED), dose per fraction, and planning target volumes were analyzed as both continuous and categorical variables at different threshold. Time was calculated from the day of surgery to the day of progression, and the hazard ratio (HR) was calculated using univariable and multivariable analysis with a Cox regression model. Data was analyzed using statistical software SPSS V26 (version 26, IBM^©^, Armonk, NY, USA).

## Results

Overall, a total of 62 patients with the diagnosis of a single BCBM who had undergone craniotomy and post-operative SRT were treated at our institution from 2010–2022. The median age was 53.5 years, most patients had a DS-GPA 2.5–4 (> 80%). Patients'characteristics are presented in Table 1. The median follow-up time of the cohort was 28 months (range, 14–43 months).

**Table 1:** Patients characteristics and clinical outcome

Variable	N = 62 patients (%)
Mean age, years (range)	53 (23–78)
Breast cancer type*	
Luminal A	5 (8%)
Luminal B	14 (22.5%)
HER- 2 positive	25 (40.3%)
Triple negative	18 (29%)
**GPA**	
0–1	0 (0%)
1.5–2	9 (14.5%)
2.5–3	23 (37%)
3.5–4	30 (48.3%)
**Size of metastases**	
0.5–1.99 cm	3 (4.8%)
2–3.99 cm	10 (16%)
4–4.99 cm	28 (45.1%)
5–6.99 cm > 7 cm	15 (24.1%) 5 (8%)
**Gross total resection**	93.5%
**Location**	
Frontal	22 (35.4%)
Parietal	13 (20.9%)
Temporal	10 (16.1%)
Occipital	8 (12.9%)
Cerebellum	8 (12.9%)
**Type of lesion**	
Solid	43 (69.3%)
Cystic	19 (30.6%)
**Time from surgery to RT in Days (mean, range)**	33(20–61)
**Adjuvant systemic therapy**	80.6%
Aromatase inhibitor + CDK4/6 inhibitor	24%
Transtuzumb + pertuzumab	8%
Transtuzumb + tucatenib + capcetabine	22%
Lapatenib + capcetabine	8%
Transtuzumab deruxtecan	4%
Transtuzumab ematnsine	8%
Single agent chemotharpy	14%
Double platinum based chemotherapy	12%
**Clinical presentation**	
Solitary brain lesion	6 (9.6%)
Oligometastatic disease (< 5 mets)	37 (59.6%)
Metastatic disease	19 (30.6%)

^*^see methods section for definition of subtype; GPA—diagnosis-specific graded prognostic assessment (Sperduto et al.,)

Breast cancer primary type was predominantly HER- 2 positive (40.3%), among them 5 (8% of total cohort) were ER/PR positive and 20 (32.3% of total cohort) were ER/PR negative; followed by triple negative breast cancer in 29%, luminal B in 22.5% and luminal A in 8% of the BCBM patient cohort. Full information of primary and BCBM receptor status was available for 51 patients, of these 63% did not have change in the receptor status, 4% gain ER, 4% gain HER2. Table 2 summarizes the primary breast cancer type and the BCBM type for the whole cohort.

**Table 2:** Primary breast cancer subtype compare to brain metastasis subtype

Brain Receptor Status versus periphery	Luminal A	Luminal B	HER- 2 Positive	Triple Negative
No change	**3**	**6**	**21**	**5**
Loss PR	**0**	**4**	**0**	**0**
Gain ER	**0**	**2**	**0**	**0**
Loss HER2	**0**	**0**	**1**	**1**
Gain HER2	**0**	**1**	**2**	**1**
Loss ER	**2**	**5**	**0**	**6**
n/a	**2**	**4**	**3**	**4**

BCBM presentation was variable, and the most frequent complaint recorded in the medical records was loss of gross limb weakness with secondary headache, aphasia and imbalance.

At presentation, 60% had oligometastatic disease, 30.4% of patients had polymetastatic disease, and 9.6% patients had a solitary brain lesion (i.e., the brain as the only site of metastases).

The most frequent location of BCBM was frontal lobe (35.4%). Solid appearance on brain MRI was more prevalent than cystic (69.4% and 30.6% respectively). Gross total resection was achieved in 93.5% of cases and 4 had sub total resection. The surgical technique used was en bloc resection; 87% had undergone en bloc resection, 9.6% piecemeal resection, and 3.2% circumferential resection.

Radiation was initiated on average 33 days from surgery with a range of 16 up to 48 days. Radiotherapy parameters are shown in Table 2. The Clinical target volume (CTV) was defined as the surgical cavity. In 79% of cases the surgical corridor was included in the CTV. CTV to planning target volume (PTV) was expanded between 2–5 mm. The average PTV was 67.1 cc (23.4–112.6). All were treated with frameless SRT, immobilization was done via SRT thermoplastic mask. SRT was performed using a Linear accelerator (LINAC). Radiation planning and delivery was via volumetric Arc radiation (VMAT) in 40 cases, in 20 cases using intensity modulated radiation (IMRT), and in two cases using 3D conformal planning (associated with treatment year).

Total dose and fractionation used are summarized in Table 3, and were according to physician preference, considering surgical cavity volume, proximity to organs. The median BED_10_ (calculated using α/β of 10 for tumor) dose of SRT was 37.5 Gy (range, 28 Gy to 59.5 Gy). Our department planning protocol for dose constraints is the TG101 report constrains^8^, all 62 treatment planning dose constraints were met.

**Table 3:** Radiation parameters for brain metastasis

Variable	Patients n – 62 (%)
PTV (median, range)	67.1 cc (23.4–112.6)
Dose (BED α/β = 10) (median, range)	37.5 Gy (28–59.5)
All radiation dosage regimen	
Total dose (Gy), n of fraction (BED α/β = 10)	
24 Gy,3 (43.2)	4 (6.4%)
24 Gy,4 (38.4)	1 (1.6%)
20 Gy,4 (30)	3 (4.8%)
25 Gy,5 (37.5)	32 (51.6%)
27.5 Gy,5 (42.6)	6 (9.6%)
30 Gy,5 (48)	11 (17.7%)
32.5 Gy,5 (53.3)	2 (3.2%)
30 Gy,6 (45)	2 (3.2%)
35 Gy,7 (59.5)	1 (1.6%)
Dose > 40 Gy	38.8%
Inclusion of corridor in PTV	79%
En bloc resection	87%
Days from surgery to RT (median, range)	28.5 (16–44)
> 28 days from RT	50%
BrainV25 (Brain-PTV) (median, range)	4.4 cc(0–9.1 cc)

PTV- Planning treatment volume, BED—Biologically Effective Dose

80.6% received systemic therapy within 3 months from radiation treatment. 31.25% of whom received HER- 2 based therapy (100% of patients with HER- 2 disease) 24% received combination of aromatase inhibitor and anti CDK4/6 (63% of luminal a and B group). And 26% received single or platinum based double chemotherapy (72.2% of triple negative group).

Local control rate was 70.9% at 12 months. The total intracranial failure (local and distant) at 12 months was 41.6%. A total of 18 (29%) patients had only local failure with 15 (out of 18, 83%) received salvage reirradiation with SRT and 3 received systemic therapy only. The median time to local progression was 13 months. Table 4 for all clinical outcomes.

**Table 4:** Outcome parameters at 12 months

Follow up months (median, range)	28 (14–43)
Local control (%)	70.9%
Time to progression (median, CI95%)	13 months (9–21)
Intracranial failure (local + distant) (%)	41.9%
Distant intracranial failure only (tumor bed controlled) (%)	12.9%
12 months overall survival (%)	80.6%
Radiation necrosis (%) Asymptomatic Symptomatic	12.9% N = 6 N = 2

A total of 6 patients (9.6%) had distant intracranial failure without local failure and all received SRT-only for the new lesions. Clinical and survival outcomes are presented in Table 3.

To determine factors associated with local control we compare different variables between patients who achieved local control at the surgical cavity site and those who did not. The median PTV was significantly larger among those who had experienced local failure (83.7 cc vs 56.2 cc, P = 0.042). The total dose delivered was lower for those with local failure with a median of 37.5 Gy for those who experienced local failure and 42.5 Gy for those who had local control (p = 0.008).

The size of the intact BCBM was significant in achieving local control: a BCBM > 5 cm was distributed differently between those with local control and local failure with 18.1% and 66.7% respectively (p < 0.001).

**Fig. 1:**
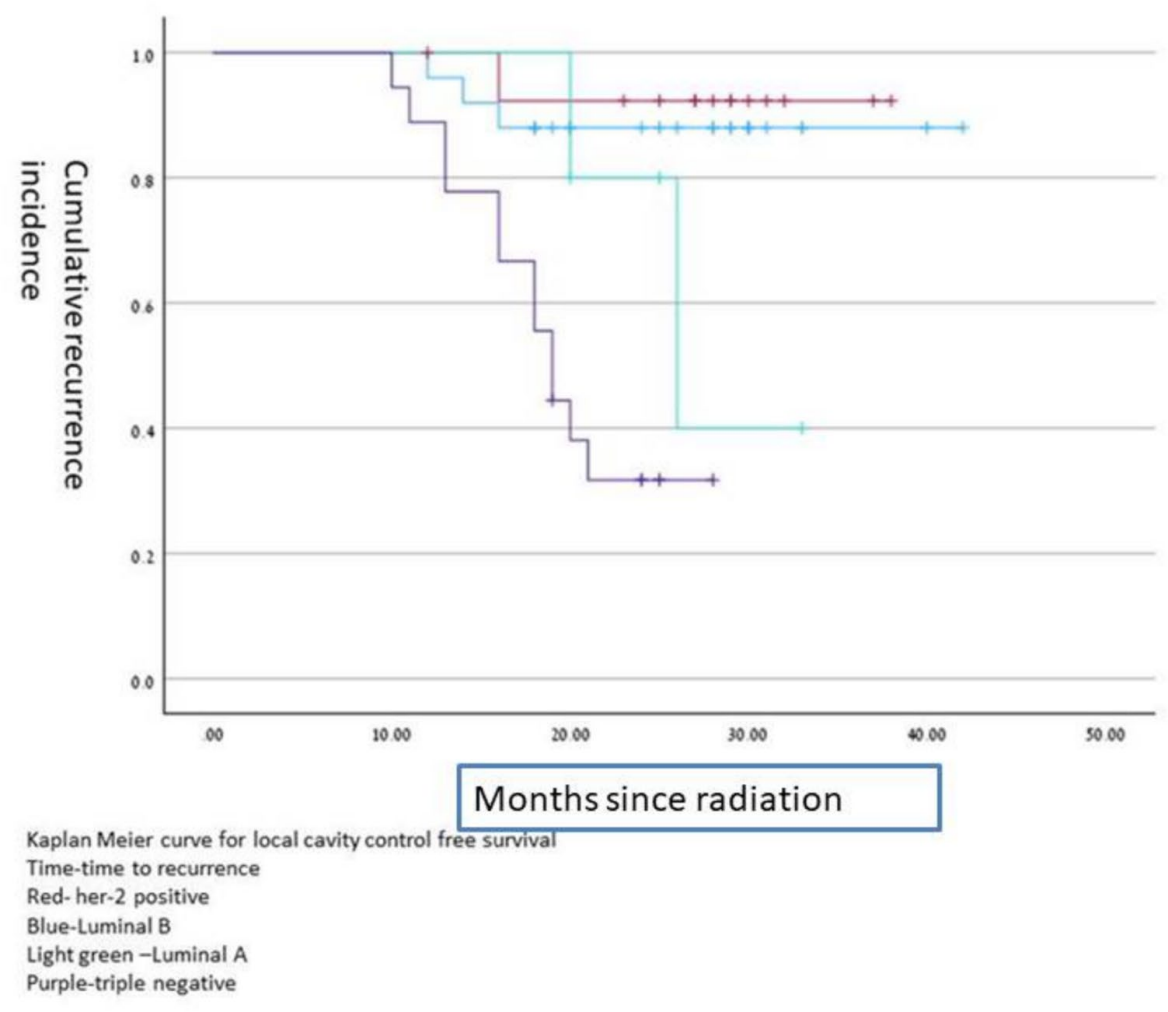
Kaplan Meier curve for surgical cavity control free survival according to brain metastasis histology

The biology of the primary breast cancer was significant, local control was significantly better in HER2 positive compared to HER2 negative (50% vs 16.1%, P = 0.015) and luminal B compare to non-luminal B (20% vs. 5%, p = 0.049) achieved local control cavity recurrence free survival time according to different histology is shown on Kaplan –Meier curves in Fig. 1.

**Fig. 2:**
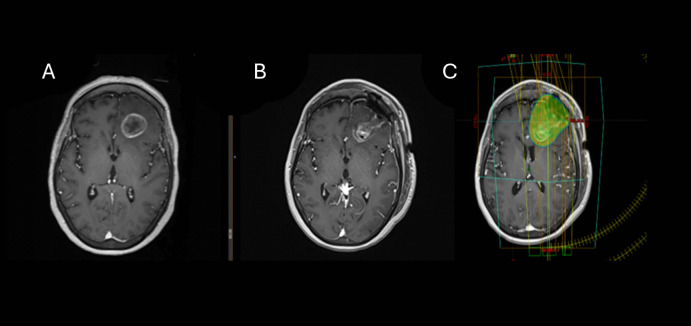
Brain MRI of a 46-year-old patient with luminal B breast cancer. Presenting symptoms of brain metastasis were headache and confusion (**A**). She underwent craniotomy for resection of a single brain metastasis at the left frontal lobe (**A**, **B**). The resection cavity was treated to a total dose of 30 Gy in 5 fractions, including the corridor (**C**). No evidence of local failure at time of last follow up

**Fig. 3:**
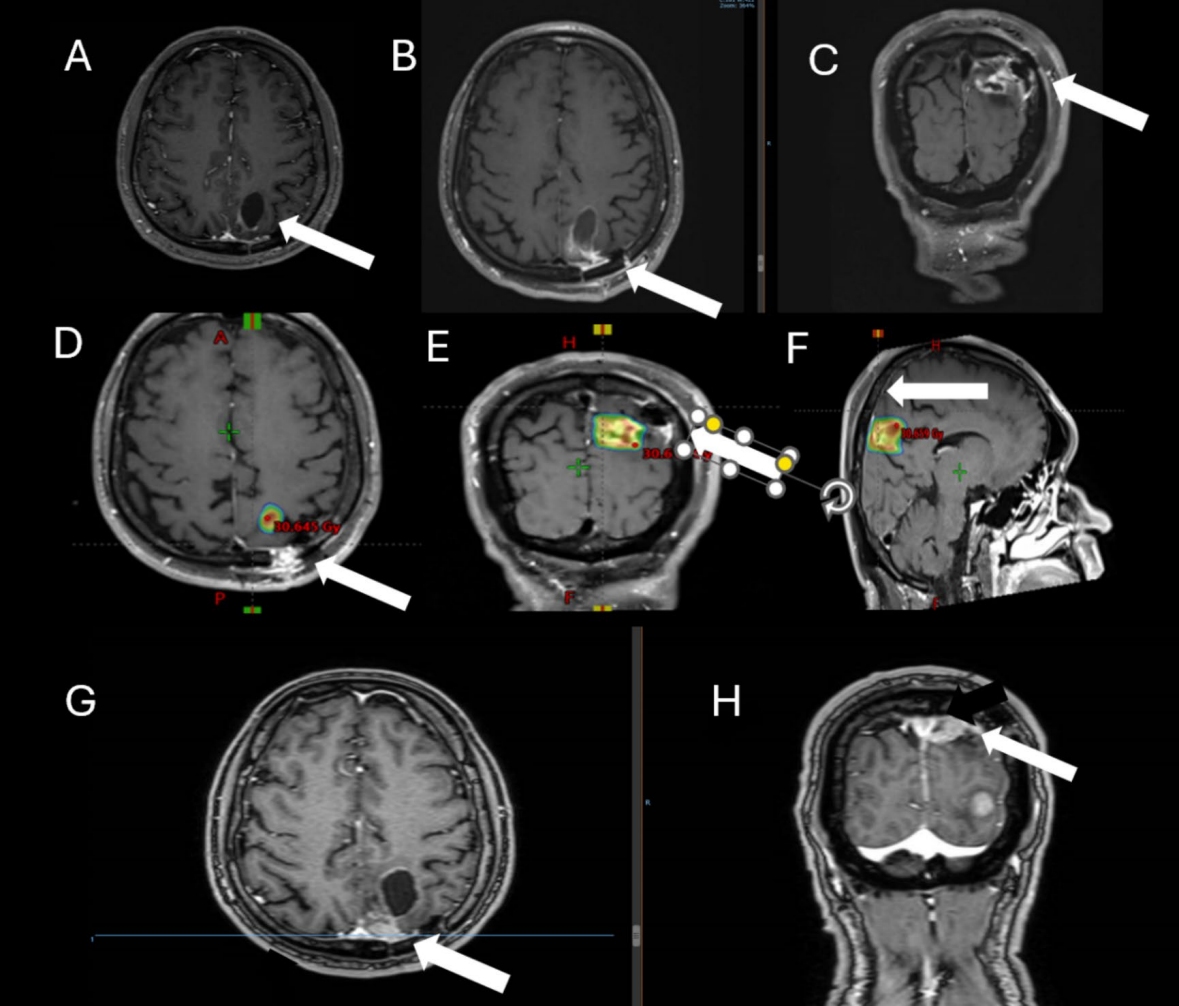
Brain MRI of a 59-year-old diagnosed with triple negative breast cancer. Presenting symptoms of brain metastasis was episode. Brain MRI showing a left parietooccipital cystic lesion (**A**). She underwent craniotomy (**B**, **C** – MRI of the surgical cavity) and the resection cavity was treated to a total dose of 25 Gy in 5 fractions not including the corridor and all surgical cavity (**D**, **E**, **F**, arrow). At 10 months she presented with local failure near the corridor (arrow) and a new brain metastasis (**G**, **H**)

Inclusion of surgical corridor within the PTV was associated with better local control (P = 0.034) (Fig. 2, 3). In addition, shorter time to radiotherapy from craniotomy with a median of 26.9 days for those who achieved local control versus 32.5 days for those had local failure (p = 0.039).

A cystic lesion associated with local failure (55.5% and 20.4% respectively P = 0.009).

The location of the surgical cavity (brain metastasis) was significance for local control, there was higher rate of failure in those with surgical cavity at cerebellum location (27.7% vs 6.8%, P = 0.039). Table 5 demonstrates all clinical variable between control lesion to recurring lesion at 12 months and odds ratio for failure.

**Table 5:** Local control vs local failure at 12 months

Variables	Local control (n = 44)	Local progression(n = 18)	HR (CI95%)	P
**Age**	53Y	53.5Y	0.97 (0.74–2.1)	0.87
**HER- 2 positive (yes)**	50%	16.7%	0.2 (0.05–0.79)	0.015
**Liminal B (yes)**	21%	5%	0.14	0.049
**Size of tumor (> 5 cm)**	18.1%	66.7%	3.4 (2.5–7.6)	< 0.001
**PTV (CC)**	56.2 CC	83.7 CC		0.042
**Dose BED (α/β = 10)** **(median, range)**	42.5 Gy (37.5–59.5)	37.5 Gy (30–48)		0.008
**BED > 40 Gy**	52.5%	16.6%	0.18 (0.049–0.79)	0.015
**Inclusion of surgical corridor (Yes)**	13.6%	38.3%	4.03 (1.1–14.5)	0.034
**Time from surgery to radiation (days median, range)**	26.9 days (18–42)	32.5 days (16–44)		0.009
**Time from surgery** ** > 28 days**	38.6%	77.8%	5.5 (1.56–19.7)	0.008
**En bloc resection**	88.6%	83.3%	1.6 (0.65–3.7)	0.43
**Type of metastases (Cystic)**	20.4%	55.5%	4.8(1.4–15.8)	0.009
**Location (cerebellum)**	6.8%	27.7%	5.2(1.1–25.4)	0.039
**Clinical presentation** **(solitary brain lesion)**	9%	11.1%	1.25(0.2–7.5)	0.8

There was no association between patient age, systemic disease, local control, or en bloc resection versus piecemeal resection.

We included all factors associates with local progression into multivariable analysis for the prediction of local progression in addition to age. We also removed PTV as it was high correlate with tumor size. Using cox regression we found that tumor size > 5 cm, HER- 2 negative and RT above 28 weeks from surgery were statistically significant for progression while BED > 40 Gy was associated with local control. Table 6.

**Table 6:** multivariable analysis for local progression

Variable	HR (CI95%), P
Age	1.003 (0.98–1.06), 0.78
**HER- 2 positive (yes)**	**0.76 (0.36–0.88), 0.032**
**Size of tumor (> 5 cm)**	**2.1 (1.7–4.6), 0.021**
**BED > 40 Gy**	**0.65 (0.45–0.89), 0.028**
Inclusion of surgical corridor (Yes)	1.3 (0.7–4.9), 0.41
**Time from surgery** ** > 28 days**	**2.7 (1.9–4.7), 0.015**
Type of metastases (Cystic)	1.5 (0.91–3.7), 0.13
Location (cerebellum)	1.75 (0.54–2.4), 0.24

^*^in bold statistically significant variables

A total of 8 cases (13%) were diagnosed with radiation necrosis on MRI. Two of whom were radiographic only and the patients were asymptomatic. The two symptomatic patients were treated successfully with dexamethasone. In the entire cohort, based on the medical records 38% reported grade 2 fatigue and 11% with grade 2 headache.

## Discussion

In this retrospective cohort we evaluate the variable impact local control among a specific group of breast cancer patients with a single BCBM undergoing craniotomy and post-operative SRT. We found that the primary tumor biology (luminal B and HER2 positive), smaller BCBM size and surgical cavity, and non-cystic lesion, higher SRT dose, higher BED, and a shorter time gap from surgery to SRT, were significant for better local control.

Notably, our local control rate is lower than previously reported studies, which showed a local control rate of 70–95% at the surgical cavity after SRT [10, 12, 20, 21, 27, 38]^.^ Several factors might be associated with inferior local control in our cohort compared to reported in trials. In our study 46% received a BED_10 Gy_ lower than 40 Gy (i.e. 25 Gy in 5 fraction) which was reported by Minnti et al., to be inferior to achieve control [25]. In addition, Soliman et al., [34] analyzed the local failures at the surgical cavity after SRT based on the data from the N107 C/CEC3 trial [NCT01372774], showed that surgical cavity size, total dose and fractionation, and correct delineation including the corridor and the surgical track are highly important for improving local control [1]. An additional factor that might be related to relatively inferior local control was the long-time gap from craniotomy to SRT in our cohort. A recent meta-analysis showed a poor local control when the time gap from craniotomy to SRT was longer than 3 weeks. The estimated 12-month control rates dropped from 87 to 61% if SRT was performed more than 3 weeks after resection [39]. A recent prospective cohort reported by Bander et al. [1]., reported that failure at the surgical cavity was highest among patients who were treated more than 30 days from craniotomy [1]. Bander et al. [1]., concluded that the optimal time to SRT is 22–30 days from craniotomy to balance between postoperative complications and local failure.

Notably, as demonstrated in our cohort, breast cancer represents a distinct entity, as shown in the breast cancer GPA [35], exhibiting a dependency on molecular subtype and potentially systemic therapies. Importantly, although none of the patients in our cohort developed leptomeningeal dissemination, breast cancer patients have a higher predisposition for leptomeningeal dissemination after SRT compared to other solid tumor brain metastases [8, 16].

It is well established in the literature that different classical sub- types of breast cancer have different biology in regards of brain metastases prevalence, pathophysiology and response to treatment [17, 31]. HER2-positive breast cancer has the inherent tendency of metastasis to the brain but because of effective systemic therapies this population have a longer survival among all breast cancer patients with brain metastases [37]. BCBM subtype switching in our cohort was lower compared to other reports [13, 15]. However, our findings do support that in case of BCBM resection evaluation of the receptors should be performed as it might be significant for systemic therapy.

Breast cancer GPA shows that having extracranial metastasis is associated with poor survival compared to solitary BCBM. Most patients in our cohort had systemic metastasis and were treated with systemic therapy after SRT, which might have contributed to a higher local control in the HER2-positive population. HER- 2 targeted therapy like transtuzumab, transtuzumab-emtansine, fam-transtuzumab-deruxtecan, lapatinib with capecitabine and tucatinib were shown to have activity in the CNS [19, 24, 36]. In the TUDEXO- 1 trial reported a rate of 83% intracranial response, therefore might be important for treating a subclinical intracranial disease and delaying the appearance of BCBM [2–4]. The improved local control in the luminal B population has been previously reported and should be further explored [34], as systemic therapy in these patients did not differ significantly from that in the luminal A-like group in our cohort. Thus, the role of systemic therapy and intrinsic radiosensitivity warrants further investigation.

The role of systemic therapy after treatment of a solitary BCBM is not well established. Disease progression in patients with a solitary BCBM occurred more intracranially than extracranially following resection [14]. Hulsbergen et al., suggested that ER positive patients with resected solitary BCBM treated with SRT, who were treated with endocrine therapy was associated with longer survival. In their cohort, HER2-targeted therapy after resection and SRT to a solitary BCBM did not show a survival benefit [14].

Our study has inherent limitations due to its retrospective nature. As our aim was to evaluate local control, we did not analyze overall survival.

Nonetheless, this study reports the outcomes of a unique population of breast cancer patients who underwent surgical resection for a single BCBM followed by postoperative SRT. This enabled us to show clinically significant factors associated with local control in breast cancer patients with BCBM, without evidence of gross tumour involving the brain. Our study emphasized the importance of proper radiation planning and the time gap from surgery to SRT for local control. Unique to BCBM, our data supports evaluation of BCBM receptor status, suggests an important role for anti-HER2 in contributing to surgical bed and intracranial control, and supports current call for research in brain metastasis to further explore unique findings associated with tumor histology and biology and treatment measures (such systemic therapy and radiation) to prevent CNS spread such as leptomeningeal disease [18].

## Data Availability

No datasets were generated or analysed during the current study.
